# Characterization of Glutamate-Mediated Hormonal Regulatory Pathway of the Drought Responses in Relation to Proline Metabolism in *Brassica napus* L.

**DOI:** 10.3390/plants9040512

**Published:** 2020-04-16

**Authors:** Van Hien La, Bok-Rye Lee, Md. Tabibul Islam, Md. Al Mamun, Sang-Hyun Park, Dong-Won Bae, Tae-Hwan Kim

**Affiliations:** 1 Department of Animal Science, Institute of Agricultural Science and Technology, College of Agriculture & Life Sciences, Chonnam National University, Gwangju 61186, Korea; hiencnsh87@gmail.com (V.H.L.); turfphy@hotmail.com (B.-R.L.); tabib_pha@hotmail.com (M.T.I.); almamun.uoda@gmail.com (M.A.M.); ghost1284@naver.com (S.-H.P.); 2 Asian Pear Research Institute, Chonnam National University, Gwangju 61186, Korea; 3 Alson H. Smith Jr. Agricultural Research and Extension Center, School of Plant and Environmental Sciences, Virginia Tech, Winchester, VA 22602, USA; 4 Biomaterial Analytical Laboratory, Central Instruments Facility, Gyeongsang National University, Jinju F52828, Korea; bdwon@gnu.ac.kr

**Keywords:** calcium signaling, glutamate, proline synthesis, redox, salicylic acid

## Abstract

Proline metabolism influences the metabolic and/or signaling pathway in regulating plant stress responses. This study aimed to characterize the physiological significance of glutamate (Glu)-mediated proline metabolism in the drought stress responses, focusing on the hormonal regulatory pathway. The responses of cytosolic Ca^2+^ signaling, proline metabolism, and redox components to the exogenous application of Glu in well-watered or drought-stressed plants were interpreted in relation to endogenous hormone status and their signaling genes. Drought-enhanced level of abscisic acid (ABA) was concomitant with the accumulation of ROS and proline, as well as loss of reducing potential, which was assessed by measuring NAD(P)H/NAD(P)^+^ and GSH/GSSG ratios. Glu application to drought-stressed plants increased both salicylic acid (SA) and cytosolic Ca^2+^ levels, with the highest expression of calcium-dependent protein kinase (*CPK5*) and salicylic acid synthesis-related *ICS1*. The SA-enhanced *CPK5* expression was closely associated with further enhancement of proline synthesis-related genes (*P5CS1, P5CS2,* and *P5CR*) expression and a reset of reducing potential with enhanced expression of redox regulating genes (*TRXh5* and *GRXC9*) in a SA-mediated *NPR1-* and/or *PR1-*dependent manner. These results clearly indicate that Glu-activated interplay between SA- and CPK5-signaling as well as Glu-enhanced proline synthesis are crucial in the amelioration of drought stress in *Brassica napus*.

## 1. Introduction

Prolonged water-deficit (e.g., drought) is considered a major climatic factor limiting plant growth and development. The decrease in water availability for transport-associated processes modifies intercellular metabolites concentration, followed by the disturbance of amino acid and carbohydrate metabolism [1,2]. An accumulation of reactive oxygen species (ROS) and/or proline is observed as a common stress response [2,3]. Indeed, rapid production of ROS (i.e., oxidative burst) is one of the earliest plant responses to stresses caused by a wide range of environmental stresses [4] and pathogen infections [5,6]. Proline accumulation has been found to be also a primary stress responsive symptom resulting from dehydration in plant tissues such as drought conditions [1,7], high salinity [8], or freezing temperature [9]. The proline pool of plant cells depends on the rate-limiting steps in proline synthesis and degradation, which are catalyzed by Δ^1^-pyrroline-5-carboxylate synthase (P5CS) and proline dehydrogenase (ProDH) [3,10,11]. Multifunctional roles of proline including in osmotic adjustment, in preventing oxidative damage, in stabilizing DNA, membranes, protein complex, as well as in providing a carbon and nitrogen source during stress have been well documented [2,7,12]. Interestingly, proline metabolism has been reported to promote mitochondrial ROS production [13]. Therefore, the modified proline metabolism by drought stress may be involved further in drought stress tolerance by regulating intracellular redox potential [10], as well as energy transfer and reducing power [3,12], which are not yet fully understood.

Increasing evidence has shown that stress responsive ROS and/or proline metabolism are regulated by hormonal signaling pathways [10,14,15]. Among these, the ABA-dependent signaling pathway has been more emphasized [16,17]. Indeed, proline accumulation is partially regulated by an ABA-dependent signaling pathway in osmotic [18] and drought stress [10]. Similarly, enhanced H_2_O_2_, as a ROS signaling from NADPH oxidase, stimulates ABA-induced proline accumulation [10,19]. Several studies have provided evidence for the ROS-mediated SA biosynthesis via Ca^2+^ signaling [14,20], as well as the proline-mediated biosynthesis of SA via NDR1-dependent signaling [21]. Recently, SA-mediated proline synthesis has been elucidated in relation to SA-dependent NPR1-mediated redox control with an antagonistic depression of ABA-signaling [10]. Furthermore, Ca^2+^-dependent protein kinases (CPKs) are now known to play a central role in innate immunity as a stress signaling by collaborating with hormonal signaling [16,20]. However, the ambivalent roles of ROS and proline in promoting stress tolerance and developing hypersensitive toxicity in connection with hormonal signaling pathway remain poorly understood.

Accordingly, the aims of the present study were to investigate the following hypotheses: (1) that exogenous Glu application would enhance proline synthesis and subsequently modify the interplay between ROS and proline metabolism in association with hormonal regulation under drought stress, and (2) that stress response and tolerance mechanisms are differently regulated by the modified hormonal state and signaling. To test these hypotheses, the effects of exogenous Glu application on drought-responsive ROS production, proline metabolism, redox state, as well as hormonal regulatory pathway were assessed to characterize the processes related to hypersensitive responses and drought tolerance mechanisms.

## 2. Results

### 2.1. Physiological Symptoms, Osmotic Potential, and Pigments

**Figure 1:**
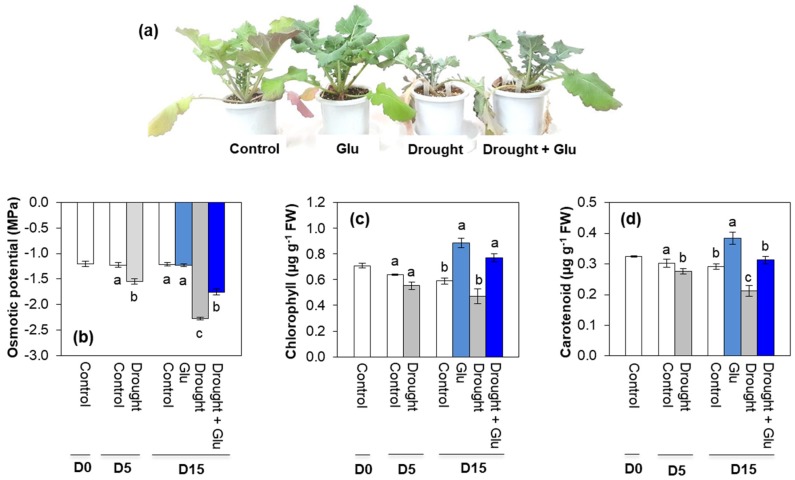
Effects of glutamate (Glu) application on (**a**) plant morphology, (**b**) osmotic potential, (**c**) chlorophyll, and (**d**) carotenoid content in the leaves of *Brassica napus* under well-watered or drought-stressed conditions. Values are represented as mean ± SE (*n* = 3). Different letters on columns indicate significant difference at *p* < 0.05 according to Duncan’s multiple range test.

Drought stress induced severe leaf wilting and reduction in leaf osmotic potential. However, drought-induced negative effects were diminished in the glutamate (Glu)-treated plants (Figure 1a,b). Drought alone treatment tended to decrease total chlorophyll and carotenoid levels, however these two photosynthetic pigments were significantly increased by Glu application after 15 d of drought. Under the well-watered conditions, exogenous Glu treatment significantly enhanced the content of these two photosynthetic pigments (Figure 1c,d).

### 2.2. Phytohormone Content and Related Gene Expression

**Figure 2:**
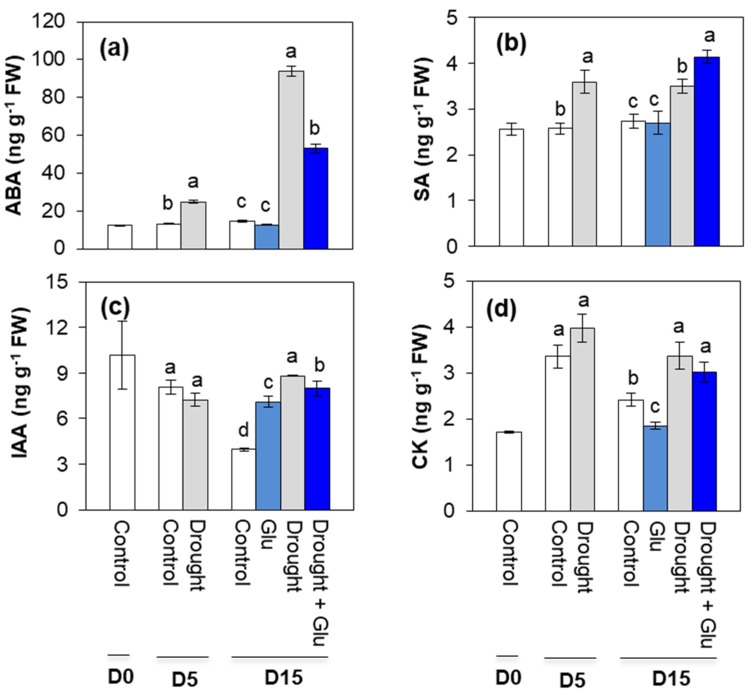
Effect of glutamate (Glu) application on endogenous phytohormone level in leaves of *Brassica napus* under well-watered or drought-stressed conditions. (**a**) Abscisic acid (ABA), (**b**) salicylic acid (SA), (**c**) indole-3-acetic acid (IAA), and (**d**) cytokinin (CK). Values are represented as mean ± SE (*n* = 3). Different letters on columns indicate significant difference at *p* < 0.05 according to Duncan’s multiple range test.

Five days of drought treatment increased endogenous ABA and SA level, but not for indole-3-acetic acid (IAA) and cytokinin (CK) (Figure 2). After 10 days of Glu application under well-watered or drought conditions (Day 15), endogenous level of ABA was remarkably increased (6.4-fold higher than control) in the drought alone treatment, whereas drought-enhanced ABA level was significantly depressed in the Drought + Glu treatment (Figure 2a).

Five days of drought treatment increased endogenous ABA and SA level, but not for indole-3-acetic acid (IAA). Drought-induced salicylic acid (SA) accumulation was further elevated in the Drought + Glu treatment (20% higher than that in drought alone), whereas no significant difference was observed in the Glu treatment under well-watered condition (Figure 2b). At day 15, endogenous IAA and CK levels significantly increased by 69% and 40%, respectively, in the drought alone, while slightly decreased for IAA or no significant change for CK level in the Drought + Glu treatment (Figure 2c,d).

**Figure 3:**
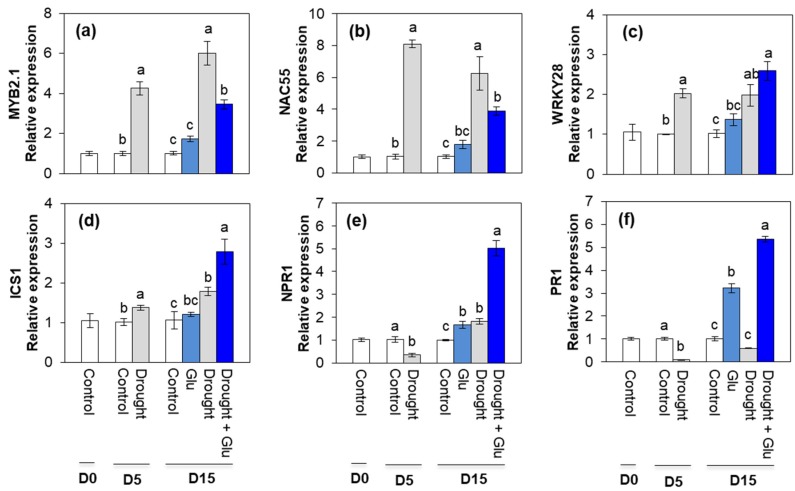
Effects of glutamate (Glu) application on the expression of ABA-responsive genes ((**a**) myb-like transcription factor (*MYB2.1*) and (**b**) NAC domain-containing protein 55 (*NAC55*)), SA-synthesis related gene ((**c**) WRKY transcription factor 28 (*WRKY28*) and (**d**) isochorismate synthase 1 (*ICS1*)), and SA-responsive genes ((**e**) non-expressor of pathogenesis-related (PR) gene (*NPR1*) and (**f**) *PR1*) in the leaves of *Brassica napus* under well-watered or drought-stressed conditions. qRT-PCR was performed in duplicate for each of the three independent biological samples. Values are represented as mean ± SE (*n* = 3). Different letters on columns indicate significant difference at *p* < 0.05 according to Duncan’s multiple range test.

Drought stress remarkably upregulated the expression of the ABA signaling-related genes, myb-like transcription factor (*MYB2.1*) and NAC domain-containing protein 55 (*NAC55*). However, drought-enhanced expression of these two genes was largely depressed by the Glu application (Figure 3a,b). In addition, expression of the SA synthesis-related genes, WRKY transcription factor 28 (*WRKY28*) and isochorismate synthase 1 (*ICS1*), were significantly upregulated by drought. A much higher expression of these genes was observed in the Drought + Glu treatment (Figure 3c,d). Expression of the SA signaling related genes, non-expressor of pathogenesis-related (PR) gene (*NPR1*) and *PR1*, were significantly depressed upon drought stress at day 5 and, then, significantly upregulated at day 15. The Drought + Glu treatment further upregulated the expression of *NPR1* and *PR1* (Figure 3e,f). No significant difference in these genes was observed in the Glu treatment under the well-watered conditions, expect for *NPR1* and *PR1* (Figure 3a–f).

### 2.3. Glutamate Receptor, ROS, Ca^2+^ Signaling, and Antioxidant Activity

**Figure 4:**
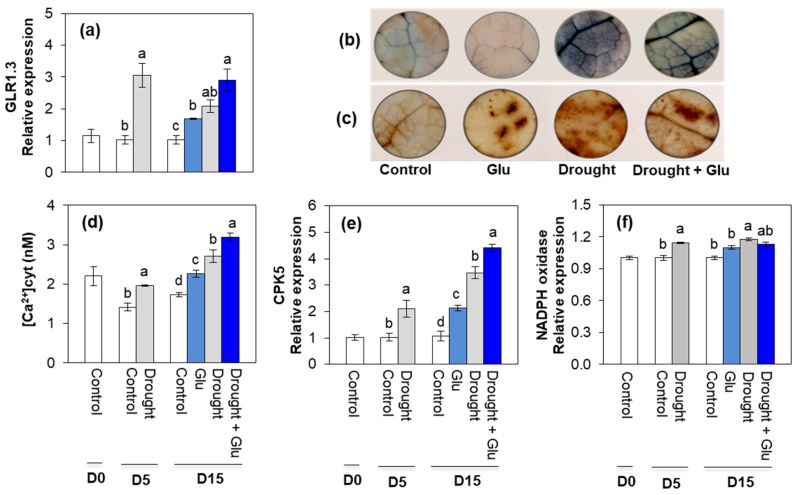
Effects of glutamate (Glu) application on (**a**) gene expression of Glutamate receptor (*GLR1.3*), (**b**) O_2_**^•−^**, and (**c**) H_2_O_2_ accumulation visualized by bark blue or brown, (**d**) cytosolic Ca^2+^ content, (**e**) calcium-dependent protein kinase 5 (*CPK5*), and (**f**) *NADPH* oxidase expression in the leaves of *Brassica napus* under well-watered or drought-stressed conditions. qRT-PCR was performed in duplicate for each of the three independent biological samples. Values are represented as mean ± SE (*n* = 3). Different letters on columns indicate significant difference at *p* < 0.05 according to Duncan’s multiple range test.

The expression of glutamate receptor, *GLR1.3*, was remarkably upregulated by drought stress. After 10 days of Glu application under well-watered or drought conditions (Day 15), Glu application upregulated *GLR1.3* by 1.8-fold and 2.9-fold, respectively, under well-watered and drought conditions compared to the levels observed in control plants (Figure 4a). A significant accumulation of ROS (O_2_**^•−^** and H_2_O_2_) was observed with in situ localization of O_2_**^•−^** and H_2_O_2_ under drought treatment, indicated by dark spots (Figure 4b,c). Cytosolic Ca^2+^ content significantly increased with drought treatment, with 56% in the drought alone treatment and 85% in the Drought + Glu treatment compared to that in the control (Figure 4d). Expression of calcium signaling-related gene, calcium-dependent protein kinase 5 (*CPK5*) was significantly induced by drought and/or Glu treatments throughout the experimental period. The greatest level was observed in the Drought + Glu treatment (Figure 4e). The expression of *NADPH* oxidase enhanced significantly only in drought alone treatment (Figure 4f). Superoxide dismutase (SOD) activity was largely increased under drought conditions, regardless of Glu treatment (Supplementary Figure S1a). The drought-induced increase in catalase (CAT) activity and its gene expression was further activated by Glu treatment (Supplementary Figure S1b,c).

### 2.4. Proline Metabolism and Transport 

**Figure 5:**
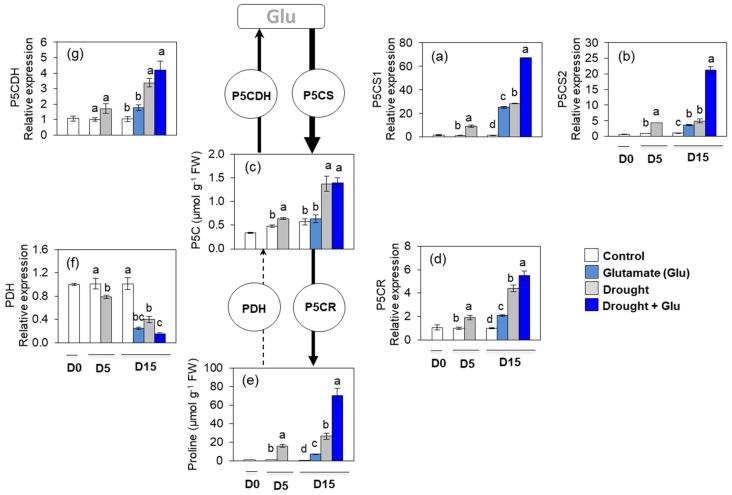
Changes in proline metabolism as affected by glutamate (Glu) application in the leaves of *Brassica napus* under well-watered or drought-stressed conditions. (**a**) Pyrroline-5-carboxylate (P5C) synthase 1 (*P5CS1*), (**b**) *P5CS2*, (**c**) P5CS content, (**d**) pyrroline-5-carboxylate reductase (*P5CR*), (**e**) proline content, (**f**) proline dehydrogenase (*PDH*), and (**g**) pyrroline-5-carboxylate dehydrogenase (*P5CDH*). qRT-PCR was performed in duplicate for each of the three independent biological samples. Values are represented as mean ± SE (*n* = 3). Different letters on columns indicate significant difference at *p* < 0.05 according to Duncan’s multiple range test.

Five days of drought significantly increased the concentration of pyrroline-5-carboxylate (P5C). At day 15, the expressions of P5C synthase 1 (P5CS1) and P5CS2 were remarkably upregulated by drought and/or Glu treatment (Figure 5a,b). Drought-induced enhancement of P5C was much higher than Glu-induced impact for both well-watered and drought conditions (Figure 5c). Drought and Glu application significantly enhanced the expression of P5C reductase (P5CR) (Figure 5d). Expression of these proline synthesis-related genes was much higher in the Drought + Glu treatment (Figure 5a,b,d). Drought induced proline accumulation throughout the experimental period, with a much greater increase in the Drought + Glu treatment (2.7-fold higher than that in the drought alone treatment; Figure 5e). The proline degradation-related genes, proline dehydrogenase (*PDH*) and pyrroline-5-carboxylate dehydrogenase (*P5CDH*), were differently expressed during the experimental period. The expression of *PDH* was largely depressed by drought and/or Glu treatments, whereas expression of *P5CDH* was significantly enhanced by the drought treatment (Figure 5f,g). Proline content in the phloem and xylem was greatly increased by drought and/or Glu treatments. The highest proline content was observed in the Drought + Glu treatment (Supplementary Figure S2a,b).

### 2.5. Redox Status and Redox Signaling Component

Both NAD(P)H and NAD(P)^+^ content tended to increase under drought condition. Drought-induced NAD(P)^+^ accumulation was significantly alleviated by Glu treatment. At day 15, the ratio of NAD(P)H to NAD(P)^+^ largely decreased in the drought alone treatment. However, drought-induced reduction was largely alleviated in the Drought + Glu treatment. Reduced glutathione (GSH) content was greatly decreased by 86.3% in the drought alone treatment compared with the control, whereas it recovered to 72.7% of that in the control in the Drought + Glu treatment. No significant difference in oxidized glutathione (GSSG) content was observed at day 15. The resulting ratio of GSH to GSSG decreased to 13% of the control, while it recovered to 76% of the control in the Drought + Glu treatment (Table 1). 

**Figure 6:**
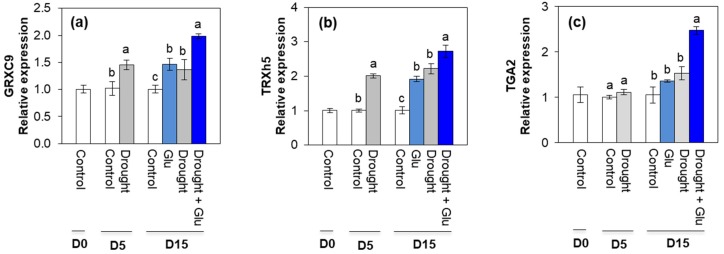
Effect of glutamate (Glu) application on the genes expression of redox signaling in leaves of *Brassica napus* under well-watered or drought-stressed conditions. (**a**) CC-type glutaredoxin 9 (*GRXC9*), (**b**) thioredoxin-h5 (*TRXh5*), and (**c**) TGA-box transcription factor (*TGA2*). qRT-PCR was performed in duplicate for each of the three independent biological samples. Values are represented as mean ± SE (*n* = 3). Different letters on columns indicate significant difference at *p* < 0.05 according to Duncan’s multiple range test.

Drought and/or Glu treatments significantly enhanced the expression of the oxidoreductase-encoding genes, CC-type glutaredoxin 9 (*GRXC9*) and thioredoxin-h5 (*TRXh5*). The expression of these two oxidoreductase-encoding genes was the highest in the Drought + Glu treatment (Figure 6a,b). The expression of TGA-box transcription factor (*TGA2*) was upregulated only in the Drought + Glu treatment (Figure 6c). 

### 2.6. Heatmap Visualization and Pearson Correlation Analysis for the Metabolites or Gene Expression

**Figure 7:**
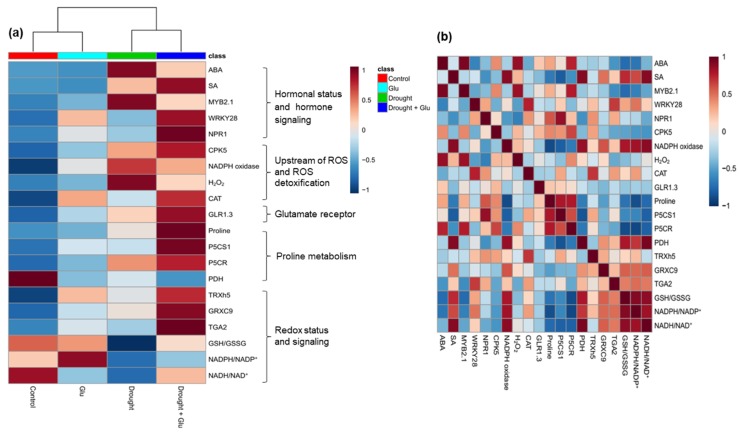
Heatmap analysis of the treatment effect and correlations among the variables measured at day 15 (after 15 d of drought, including 10 d of glutamate application). (**a**) Heatmap comparing the changes in the identified metabolites or gene expression levels in the leaves of control or glutamate (Glu)-treated plants under well-watered or drought-stressed conditions. The normalization procedure consisted of mean row-centering with color scales. (**b**) Heatmap showing the correlations among the identified metabolites or gene expression levels. Correlations coefficients were calculated based on Pearson’s correlation. Red indicates a positive effect, whereas blue indicates a negative effect. Color intensity is proportional to the correlation coefficients.

To further clarify the metabolites or gene expression levels affected by the drought-stress and/or Glu treatments, the resulting data of hormones, ROS, upstream ROS signal, glutamate receptor, proline metabolism, redox status, and their signaling were visualized by heatmap and Pearson correlation coefficients (Figure 7). The drought exhibited notable influences on the increase of endogenous ABA level and ABA signaling gene *MYB2.1*, H_2_O_2_, NADPH oxidase as well as on the loss of reducing potential NAD(P)H/NAD(P)^+^ and GSH/GSSG. These drought effects were alleviated by Glu application, resulting in an increase in SA and its synthesis or signaling gene (*NPR1* or *WRKY28*, respectively), CPK5, reducing potential, and proline synthesis (Figure 7a). Proline was positively correlated with SA level, glutamate receptor GLR1.3, and the redox-signaling genes *TRXh5* and *GRXC9* in a positive relation with the expression of the SA-signaling regulatory genes *NPR1* and *CPK5* (Figure 7b). 

## 3. Discussion

The accumulation of proline in plants tissue has been commonly observed under abiotic and biotic stress conditions. This stress response is thought to function as a protective mechanism in stressed plants [20,22]. However, proline metabolism is responsible for stress-induced ROS production and is, subsequently, involved in the hypersensitive response of plants [13]. Therefore, determining the thresholds of regulatory mechanisms at which proline metabolism switches from hypersensitive responses to stress resistance (or vice versa) would provide valuable insight into the underlying mechanisms of plant stress responses. Accordingly, one of the aims of the present study was to test the hypothesis that exogenous Glu would accelerate proline synthesis, because proline is mainly synthesized from Glu under drought conditions [12] and because the early Glu-responsive genes encode membrane receptors, protein kinase/phosphatases, Ca^2+^ signaling, and transcription factors [23]. The present study, thus, assessed preferentially the effect of Glu-responsive proline metabolism on drought symptom development. 

In the present study, 5 days of drought induced an accumulation of both ROS and proline, which has been commonly observed in drought-stressed plants [3,10,24], and another 10 d of drought (15 d in total) provoked severe drought symptoms, such as leaf wilting and reduced leaf osmotic potential (Figure 1a,b). These drought-induced hypersensitive responses were accompanied by enhanced ROS accumulation (Figure 4b,c) and reduced reducing potential (Table 1). Severe drought symptoms in drought alone was concomitant with the highest ABA accumulation and expression of ABA-related genes (Figure 2a and Figure 3a,b). ABA has been reported to stimulate a signaling pathway that triggers ROS production, which in turn induces increases in cytosolic Ca^2+^ [17]. Indeed, drought-induced ABA-mediated ROS accumulation was concomitant with increased levels of NADPH oxidase (Figure 4f), accompanied by cytosolic Ca^2+^ (Figure 4d) and CPK5 (Figure 4e), which is consistent with the findings of previous studies [11,16,25]. ROS (mainly H_2_O_2_) accumulation that is accompanied by redox changes might directly or indirectly involve regulating the transcription of proline biosynthesis [10,11]. In the present study, a significant accumulation of proline with enhanced expression of proline synthesis-related genes was observed in drought-stressed plants, regardless of Glu treatment (Figure 5). Previous studies have also reported ABA-induced proline accumulation [19]. The simultaneous accumulation of ROS and ABA has been postulated as a key aspect of cross-tolerance [19]. Furthermore, the interplay between ABA, ROS, and proline has been suggested to function as an integrative process in regulating water stress responses and signal transduction pathways [13,17,19]. However, in the present study, the drought-induced ABA-responsive enhancement of ROS and proline was a hypersensitive response that included the expression of severe symptoms, whereas the negative symptom induced by drought was significantly alleviated in the Drought + Glu treatment, despite the additional accumulation of ROS and proline (Figure 1, Figure 4 and Figure 5). It is, therefore, tempting to characterize the plant immune and stress-signaling networks that trigger appropriate and diverse downstream responses to drought stress. Of the many networks involved in responses to drought stress, the present study focused on Ca^2+^-dependent protein kinases (CPKs) because recent studies have highlighted the roles of CPK-signaling pathways in plant immune and stress responses [16,25,26]. In the proposed model for interactions between ROS and Ca^2+^ signaling [16,25], CPKs, upon activation by the Ca^2+^ flux, together with a mitogen-activated protein kinase (MAPK), trigger the expression of immunity-related genes [25]. Meanwhile, several protein kinases, including CPKs, enhance the activity of Rbohs (i.e., NADPH oxidase), thereby promoting the generation of apoplastic ROS [16,27]. In the present study, the drought-stress treatment induced increases in glutamate receptor GLR1.3 (Figure 4a), cytosolic Ca^2+^ (Figure 4d), and *CPK5* expression (Figure 4e), regardless of Glu treatment. Boudsocq and Sheen (2013) reported that the signal through ABA synthesis activates CPKs, which regulate ROS and proline accumulation, water transport (e.g., aquaporin) as well as expression of related genes. Indeed, in this study, the enhanced CPK5 expression in the treatment drought alone was concomitant with an accumulation of ROS (Figure 4b,c) and proline (Figure 5e), accompanied by the highest ABA level and expression of ABA-signaling genes (Figure 2a and Figure 3a,b). In rice, CPKs have been reported to enhance salt-stress tolerance by regulating ROS homeostasis through the induction of ROS scavenging genes (*APX2/APX3*) and the suppression of the NADPH oxidase gene, *Rboh1* [28]. However, in the present study, drought-enhanced ABA-responsive CPK5 was not observed to either suppress NADPH oxidase or scavenge ROS (Figure 4). Interestingly, the Drought + Glu treatment further upregulated *CPK5* expression, thereby increasing both endogenous SA and the expression of SA synthesis- and signaling-related genes (*ICS1* and *NPR1*, respectively), with antagonistic depression of ABA level (Figure 2a) and the expression of ABA-signaling genes (*MYB2.1* and *NAC55*; Figure 3a,b). The increased SA and SA-related gene expression, which coincided with exogenous Glu-enhanced-CPK5, significantly reduced the accumulation of ROS (Figure 4b,c) and increased the accumulation of proline (Figure 5e), thereby alleviating the negative symptoms of drought stress (Figure 1). It is worth noting that there was a remarkable difference in the drought symptoms between the Drought alone and Drought + Glu treatments (Figure 1a), even though plants in both treatments exhibited a significant accumulation of ROS and proline, as well as enhanced cytosolic Ca^2+^ and *CPK5* expression. The difference of drought symptom development (Figure 1) with distinct changes in the hormonal balance and gene expression of the two treatment groups (Figure 2 and Figure 3) lead us to further investigate the hormonal regulatory pathways involved in the integrative process of stress response and tolerance.

Several reviews have documented that ROS and proline that is accumulated in response to stress stimuli function as signaling molecules, with possible interactions with phytohormonal signaling in metabolic regulatory pathways [3,12,13,14]. In the present study, the simultaneous and significant accumulation of ROS and proline, accompanied by elevated cytosolic Ca^2+^ and *CPK5* expression, was observed under drought stress, regardless of Glu treatment. However, the pattern of ROS and proline, as well as cytosolic Ca^2+^ and *CPK5* expression followed by ABA-dependent in the treatment Drought alone, while SA-dependent manner in the treatment Drought + Glu (Figure 2, Figure 4 and Figure 5e). Furthermore, drought-induced proline was much more increased in the treatment Drought + Glu, accompanied by further enhancements of proline synthesis-related genes (*P5CS* and *P5CR*) and depression of proline degradation-related gene (*PDH*; Figure 5) expression. The accumulation of proline in response to exogenous Glu treatment, along with the additional activation of Ca^2+^ and CPK5, was induced in a SA-dependent manner (Figure 2b and Figure 4d,e). The Ca^2+^-binding transcription factor CBP60g regulates the transcription of SA biosynthesis genes (e.g., *ICS1/SID2;* [29,30])*,* thereby providing a venue for the Ca^2+^ signal to activate the WRKY28 transcription factor (Figure 3c) in SA production. Indeed, the highest expression levels of *ICS1*, *NPR1*, and *PR1* in the Drought + Glu plants were consistent with the highest proline level and enhanced expression of proline synthesis-related genes (Figure 3d–f and Figure 5), as well as with the downregulation of ABA (Figure 2a). Similarly, Chen et al. (2011) [21] reported that exogenous proline significantly induced intracellular Ca^2+^ accumulation and Ca^2+^-dependent ROS production, thereby enhancing SA synthesis. The results of several other studies have supported the interplay between SA and proline in regulating stress responses, e.g., proline-activated SA-induced protein kinase SIPK [31], involvement of SA in exogenous proline-induced salt resistance [21], and proline-mediated drought tolerance [10]. Furthermore, elevated SA levels suppressed ROS production in the present study (Figure 4b,c), potentially through a feedback loop for O_2_^•−^ [32] and the enhanced activation of CAT for scavenging H_2_O_2_ (Supplementary Figure S1b). Indeed, SA-activated CAT [10,14] and Ca^2+^-dependent CAT activation [33] have been reported previously. In addition, as far as we know, this study provides the first report of exogenous Glu-increased proline loading to both the xylem and phloem (Supplementary Figure S2). Given that glutamate triggers long-distance, Ca^2+^-based plant defense signaling, it is reasonable to conclude that the Glu-mediated overproduction of proline could be responsible for SA production and the activation of SA-signaling and involve also in activation of Ca^2+^-mediated signaling, thereby functioning as a crucial regulatory pathway of stress tolerance. However, the mechanism by which proline- or SA-elicited ROS signals activate CPK5 remains unclear and requires further investigation. 

Calcium-mediated signaling that occurs after the accumulation of SA has been reported to contribute to the regulation of defense-related gene expression. The interaction of Ca^2+^ is enhanced by the binding of Ca^2+^ to leucine zipper transcription factor TGA [34], which interacts with NPR1, a critical transcription cofactor in SA perception and the SA-mediated transcriptional regulation of PR1 through NPR1 [14,20], thereby providing a possible SA-mediated option to regulate stress tolerance reactions. In the present study, exogenous Glu-responsive, SA-mediated *NPR1* and *PR1* expression was consistent with the expression of TGA2 and CPK5, which was highest in the Drought + Glu plants (Figure 3e–f, Figure 4e and Figure 6c). Moreover, a synergistic and significant interaction between proline and SA for SA-transduction signaling (*NPR1* and *PR1*) was also observed in the Drought + Glu (Figure 3e–f and Figure 5e). Increasing evidence demonstrates that *NPR1* is the first redox sensor to be described for SA-regulated genes and that *NPR1* is the master co-activator of *PR1* [10,33,35,36]. Over-produced proline also activated the SA-signaling pathway but not the JA-signaling pathway [21].

Given that proline metabolism is directly involved in the control of NAD(P)^+^/NAD(P)H redox balance [3,37]. A significant recovery of reducing potential GSH/GSSG and NAD(P)H/NAD(P)^+^ ratios in the treatment Drought + Glu (Table 1) would be closely related with Glu-enhanced proline synthesis, as part of SA-mediated redox regulation. Indeed, in the Drought + Glu treatment, the oxidoreductase-encoding genes *TRXh5* and *GRXC9* were upregulated in a SA-mediated, NPR1-dependent manner (Figure 3e and Figure 6). These genes are essential for redox control in SA-mediated transcriptional responses [14,33,36]. Therefore, the results of both the present study and previous reports [10,20,33] provide evidence that SA-mediated, NPR1-dependent transcriptional responses, which may interact with proline metabolism, are integrative cellular redox regulation processes that promote PR1 induction. 

**Figure 8:**
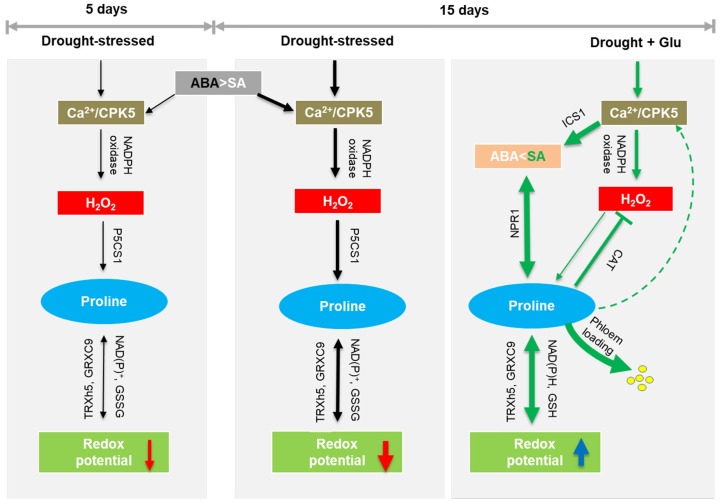
Proposed model for glutamate-mediated hormone antagonism, proline synthesis, and redox modulation under drought and/or glutamate treatment. Black arrows represent the ABA-dependent pathway of response to drought, and green arrows represent the glutamate-mediated SA pathway under drought. Red or blue arrows indicate the decrease or increase of redox potential. The thickness of the arrow expresses the strength of induced or depressed response.

The results of the heatmap and Pearson correlation analysis (Figure 7) provide a basis for a working model of the signaling pathway that is activated by exogenous Glu (Figure 8). The resulting data showed that the impacts of drought on SA- and CPK5-signaling as well as proline synthesis were higher than that of Glu under well-watered condition. Therefore, the model for Glu-mediated modulation only under drought condition was presented. In summary, the drought-induced negative stress responses were largely alleviated by exogenous Glu-induced, SA-mediated modulations that were characterized by (1) antagonistic depression of ABA-dependent metabolic and signaling pathways, (2) synergetic interaction of CPK5-mediated SA induction and proline synthesis, and (3) SA-mediated NPR1-dependent redox regulation.

## 4. Materials and methods

### 4.1. Plant Growth and Treatment

*Brassica napus* (cv. Pollen) seeds were germinated in the bed soil mixed with soil, perlite, and cocopeat (50:40:10, w/w/w) in a tray. The soil used for the pot experiment was air-dried, sieved, and then moistened to −30 kPa water potential. The treated soil was sterilized by incubating at 25 °C for 48 h. Upon reaching the four-leaf stage, seedlings were transplanted in 2-L pots that contained a 70:30 (w:w) mixture of soil and perlite, and grown with 100 mL nutrients solution [4]. At the 6–7 leaves stage, plants were selected by morphological similarity and divided into two groups for the drought treatment. One group of well-watered plants was daily irrigated with 200 mL of water, while the other group of drought-stressed plants with 20 mL. After 5 days of drought treatment, both the well-watered and drought-treated groups were divided into two subgroups of glutamate application that were applied without or with 20 mM glutamate. Glutamate application was done on the basis of preliminary test referring to the previous study [23]. Briefly, 2 mL of 20 mM glutamate was applied just after daily irrigation through four porous plastic tubes, which were put 5 cm below the soil surface in each pot, to drive directly the applied water and glutamate to the root zone. Thus, the experiment consisted of four treatments: well-watered (Control), Glu application under well-watered (Glu), drought alone (Drought), and Glu application under drought condition (Drought + Glu). The plants were grown in a greenhouse with day/night mean temperature of 27/20 °C and relative humidity of 65%/85%. Natural light was supplemented by metal halide lamps that generated 200 μmol photons m^−2^ s^−1^ at the canopy height for 16 h per day. The sampling was conducted at the day of drought treatment (D0), after 5 days of drought treatment (D5), and after 10 days of Glu application (D15), respectively.

### 4.2. Osmotic Potential and Measurement of Photosynthetic Pigment Content 

For the measurement of osmotic potential, fresh leaves were frozen in liquid nitrogen and then allowed to thaw, followed by centrifugation at 13,000× *g* for 15 min. The collected sap was used for measuring osmolality by using a vapor pressure osmometer (Wescor 5100; Wescor Inc., Logan, UT). For total chlorophyll and carotenoid content, fresh leaves (100 mg) were immersed in 10 mL of 99% dimethyl sulfoxide following the previous method [38]. After 48 h, the absorbance of the supernatants was read at 480 and 510 nm for carotenoid, and 645 and 663 nm for total chlorophyll by using a microplate reader (Synergy H1 Hybrid Reader; Biotek, Korea). The calculation of two pigments was as follows: total Chl (µg) = 20.2 A645 + 8.02 A663 and carotenoid (µg) = 7.6 A480 + 1.49 A510.

### 4.3. Determination of ROS Production and Antioxidative Enzymes Activity 

For the visualization of H_2_O_2_ and O_2_^•−^, leaf discs were stained with 3,3′-diaminobenzidine (DAB) and nitroblue tetrazolium (NBT), respectively, as described previously [6,7]. The activities of superoxide dismutase (SOD; EC 1.15.1) and catalase (CAT; EC 1.11.1.6) were determined using the method of Lee et al. (2013). One unit of SOD enzyme activity was defined as the amount of enzyme required to inhibit 50% of the NBT photoreduction observed in negative control reactions. One unit of CAT enzyme activity was defined as the amount of enzyme required to degrade 1 mM H_2_O_2_ min^−1^.

### 4.4. Measurement of Cytosolic Ca^2+^ Concentration 

Cytosolic Ca^2+^ levels were estimated using aequorin luminometry detection [39] with some modifications. Briefly, 200 mg fresh leaves were extracted in a buffer solution containing 1 mM KCl, 1 mM CaCl_2_, and 10 mM MgCl_2_, adjusted pH to 5.7 using Tris-base, and centrifuged at 12,000× *g* for 10 min. One hundred micro liters of supernatant was incubated with 1 µL of 0.1 mM coelenterazine-h in a 96-well plate for 30 min to facilitate binding between coelenterazine-h (Sigma) and aequorin. After incubation, an equal volume of 2 M CaCl_2_, which was dissolved in 30% ethanol (v/v), was added to discharge the remaining aequorin. Calcium concentration was determined by luminescence, according to Knight et al. (1996) [40].

### 4.5. Determination of Proline and Δ^1^-pyrroline-5-carboxylate Content

For the determination of proline and pyrroline-5-carboxylate (P5C) content, fresh leaf (200 mg) was homogenized in 3% sulfosalicylic acid and centrifuged at 13,000× *g* for 10 min. The supernatant collected was used for further analysis. For proline analysis according to the method described by Bates et al. (1973) [41], the resulting supernatants were mixed with ninhydrin solution containing acetic acid and 6 M H_3_PO_4_ (v/v, 3:2) and boiled at 100 °C for 1 h. Then, toluene was added to the mixture, which was incubated for 30 min. The absorbance was determined at 520 nm and quantified proline concentration as described previously [24]. 

P5C content was determined according to method described by Mezl and Knox (1976) [42]. The supernatants were mixed with 10 mM of 2-aminobenzaldehyde dissolved in 40% ethanol. Then, the mixture was incubated at 37 °C for 2 h to develop the yellow color. The absorbance was measured at 440 nm and calculated by using an extinction coefficient 2.58 mM^−1^ cm^−1^.

### 4.6. Collection of Phloem Exudate and Xylem Sap 

Phloem exudates were collected in ethylenediaminetetraacetic acid (EDTA) using the facilitated diffusion method, as described previously [24]. The fourth fully extended leaf was cut and immediately rinsed in 20 mM EDTA solution (pH 7.0) for 5 min. The leaf was then transferred to a new tube containing 5 mM EDTA solution and kept for 6 h in a growth chamber with 95% relative humidity under dark conditions. Xylem sap was collected by a vacuum-suction technique [43]. Both the phloem exudates and xylem sap were stored at −20 °C for further analysis. 

### 4.7. Measurement of Glutathione and Pyridine Nucleotides

For the extraction of glutathione, 200 mg fresh leaves were homogenized in 5% 5-sulfosalicylic acid and centrifuged at 12,000× *g* for 10 min. The glutathione content of the resulting supernatants was then determined by microplate assay using the GSH/GSSG Kit GT40 (Oxford Biomedical Research, Inc.). The contents of oxidized and reduced pyridine nucleotides were measured as described previously [10]. 

### 4.8. Phytohormone Analysis

Quantitative analysis of phytohormones in leaf tissue was performed by a high-performance liquid chromatography-electrospray ionization tandem mass spectrometry (HPLC–ESI–MS/MS) [10,44]. Brief, 50 mg of fresh leaves in a 2-mL tube was frozen in liquid nitrogen and ground using a Tissuelyser II (Qiagen). The ground sample was extracted with 500 μL of extraction solvent, 2-propanol/H_2_O/concentrated HCl (2:1:0.002, v/v/v). Dichloromethane (1 mL) was added to the supernatant, and this was then centrifuged at 13,000× *g* for 5 min at 4 °C. The lower phase, which was poured into a clean screw-cap glass vial, was dried under nitrogen and dissolved in pure methanol. The completely dissolved extract, ensured by vortexing and sonicating, was transferred to a reduced volume liquid chromatography vial. Hormones were analyzed by a reverse phase C18 Gemini high-performance liquid chromatography (HPLC) column for HPLC–ESI–MS/MS analysis. The chromatographic separation of hormones and its internal standard from the plant extracts was performed on an Agilent 1100 HPLC (Agilent Technologies), Waters C18 column (15,092.1 mm, 5 l m), and API3000 MSMRM (Applied Biosystems), using a binary solvent system comprising 0.1% formic acid in water (Solvent A) and 0.1% formic acid in methanol (Solvent B) at a flow rate of 0.5 ml/min.

### 4.9. RNA Extraction and Quantitative Real-Time PCR Analysis

Total RNA was isolated from 200 mg fresh leaf using an RNAiso Plus (Takara, DALIAN), and cDNA was synthesized using the GoScript Reverse Transcription System (Promega, United States). Gene expression was quantified using a light cycle real-time PCR detection system (Bio-Rad, Hercules, CA, USA) with SYBR Premix Ex Taq (Takara, DALIAN, Japan). The PCR reactions were performed using the following conditions: 95 °C for 5 min; and then followed by 45 cycles of 95 °C for 30 s, 55–60 °C for 30 s, and 72 °C for 30 s; and a final extension of 72 °C for 5 min. The qRT-PCR was performed using gene-specific primers (Supplementary Table S1). The qPCR reactions were performed in triplicate for each of three independent samples, and the relative expression levels of the target genes were calculated from threshold values (Ct), using the 2^−∆∆CT^ method [45] and the actin gene as an internal control. 

### 4.10. Statistical Analysis

The present study used a completely randomized design with three replicates for each treatment and sampling date. Analysis of variance (ANOVA) was applied to all data, and Duncan’s multiple range test was used to compare the means of separate replicates for each sampling time. All statistical tests were performed using SAS 9.1 (SAS Institute, Inc., 2002-2003), and differences at *p* < 0.05 were considered significant. The heatmap, correlation coefficient, and pathway impact analyses were performed using MetaboAnalyst 3.0 (http://www.metaboanalyst.ca).

## Figures and Tables

**Table 1:** Changes in redox status as affected by glutamate (Glu) application in the leaves of *Brassica napus* under well-watered or drought-stressed conditions.

Treatments	Days after Treatment								
	0			5			15		
**Reduced**	**NADPH**	**NADH**	**GSH**	**NADPH**	**NADH**	**GSH**	**NADPH**	**NADH**	**GSH**
Control	2.65 ± 0.83	5.39 ± 0.48	61.51 ± 4.81	2.62 ± 0.17 ^b^	5.58 ± 0.44 ^a^	58.58 ± 2.01 ^a^	2.32 ± 0.25 ^c^	4.44 ± 0.33 ^c^	53.20 ± 3.26 ^a^
Glu	-	-	-	-	-	-	5.28 ± 0.39 ^a^	5.07 ± 0.40 ^bc^	60.35 ± 5.00^a^
Drought	-	-	-	3.48 ± 0.25 ^a^	5.79 ± 0.12 ^a^	15.77 ± 0.21 ^b^	3.78 ± 0.10 ^b^	5.37 ± 0.03 ^b^	7.27 ± 0.12 ^c^
Drought + Glu	-	-	-	-	-	-	4.84 ± 0.04 ^b^	6.56 ± 0.03 ^a^	38.67 ± 1.87 ^b^
**Oxidized**	**NADP^+^**	**NAD^+^**	**GSSG**	**NADP^+^**	**NAD^+^**	**GSSG**	**NADP^+^**	**NAD^+^**	**GSSG**
Control	8.86 ± 0.06	6.81 ± 0.06	2.30 ± 0.05	9.62 ± 0.59 ^b^	6.82 ± 0.30 ^b^	2.62 ± 0.05 ^b^	7.23 ± 1.05 ^d^	6.01 ± 0.22 ^c^	2.45 ± 0.09 ^a^
Glu	-	-	-	-	-	-	14.36 ± 0.61 ^c^	8.53 ± 0.25 ^b^	3.10 ± 0.25 ^a^
Drought	-	-	-	17.80 ± 1.00 ^a^	9.10 ± 0.17 ^a^	3.23 ± 0.13 ^a^	24.57 ± 0.30 ^a^	9.89 ± 0.07 ^a^	2.69 ± 0.22 ^a^
Drought + Glu	-	-	-	-	-	-	21.36 ± 0.52 ^b^	9.51 ± 0.17 ^a^	2.47 ± 0.22 ^a^
**Ratios**	**NADPH/** **NADP^+^**	**NADH/** **NAD^+^**	**GSH/** **GSSG**	**NADPH/** **NADP^+^**	**NADH/** **NAD^+^**	**GSH/** **GSSG**	**NADPH/** **NADP^+^**	**NADH/** **NAD^+^**	**GSH/** **GSSG**
Control	0.41 ± 0.02	0.79 ± 0.07	26.65 ± 1.98	0.26 ± 0.00 ^a^	0.81 ± 0.03 ^a^	25.94 ± 1.02 ^a^	0.27 ± 0.02 ^b^	0.74 ± 0.06 ^a^	21.70 ± 0.67 ^a^
Glu	-	-	-	-	-	-	0.37 ± 0.02 ^a^	0.60 ± 0.04 ^ab^	19.58 ± 1.20 ^ab^
Drought	-	-	-	0.21 ± 0.01 ^a^	0.64 ± 0.02 ^b^	4.92 ± 0.25 ^b^	0.15 ± 0.03 ^c^	0.54 ± 0.00 ^b^	2.75 ± 0.21 ^c^
Drought + Glu	-	-	-	-	-	-	0.21 ± 0.00 ^bc^	0.69 ± 0.01 ^ab^	15.96 ± 1.36 ^b^

Reduced form of nicotinamide adenine dinucleotide (phosphate), NAD(P)H; oxidized form of nicotinamide adenine dinucleotide (phosphate), NAD(P)+; reduced form of glutathione, GSH; oxidized form of glutathione, GSSG. NAD(P)H and NAD(P)^+^ contents are shown as nmol g^−1^ fresh weight. GSH and GSSG contents are shown as μmol g^−1^ fresh weight. Values are mean ± SE for *n* = 3. Different lowercase letters in a column indicate significant differences at *p* < 0.05 according to Duncan’s multiple range test.

## Supplementary Materials

The following are available online at https://www.mdpi.com/2223-7747/9/4/512/s1, Table S1: (Oligonucleotide primer sequences used for quantitative real-time PCR) and Figure S1: (Effect of glutamate (Glu) application on the antioxidative enzymes activity and catalase (*CAT)* gene expression in the leaves of *Brassica napus* under well-watered or drought-stressed conditions), Figure S2: (Effect of glutamate (Glu) application on the proline content in phloem and xylem in *Brassica napus* under well-watered or drought-stressed conditions).
