# Supplementary material for: Characterization of Glutamate-Mediated Hormonal Regulatory Pathway of the Drought Responses in Relation to Proline Metabolism in Brassica napus L
Source: Plants (Basel). 2020 Apr 16;9(4):512. doi: 10.3390/plants9040512 (PMC7237994; doi:10.3390/plants9040512)
Supplement: Supplementary file 1 [file plants-09-00512-s001.zip › Figures_Supporting figures.pdf]

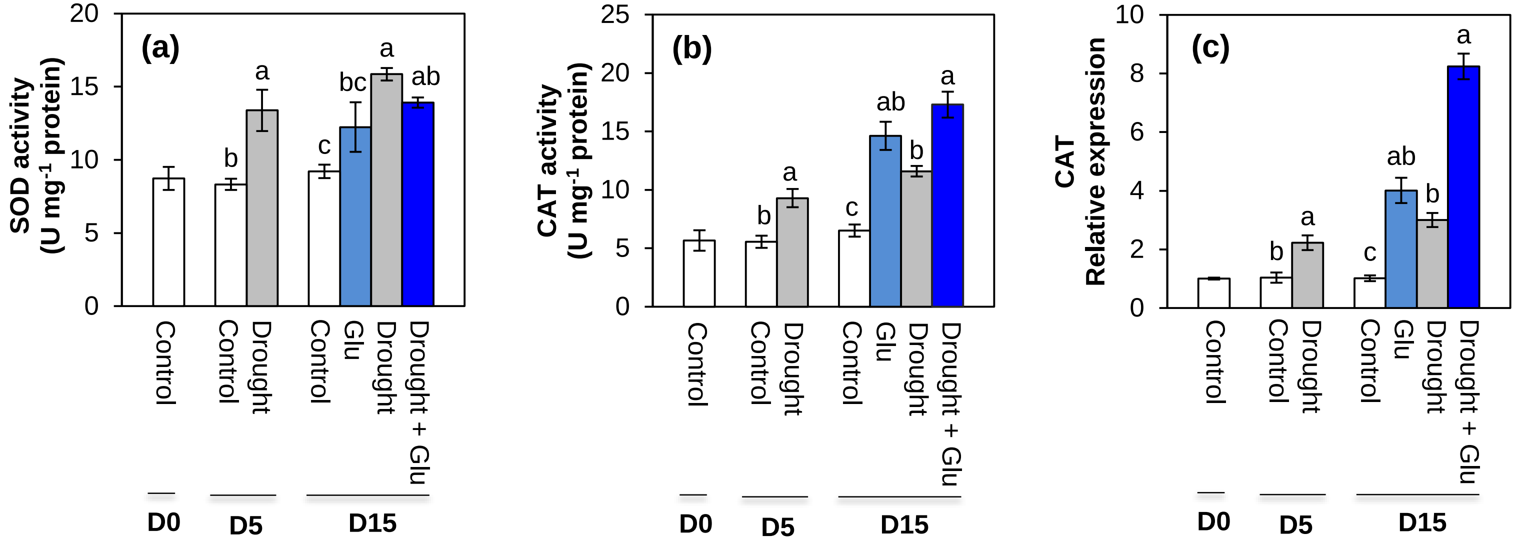

Figure S1. Effect of glutamate (Glu) application on the antioxidative enzymes activity and catalase (*CAT)* gene expression in the leaves of *Brassica napus* under well-watered or drought-stressed condition. (a) Superoxide dismutase (SOD) and (b) CAT activity and (c) *CAT* gene expression. qRT-PCR was performed in duplicate for each of the three independent biological samples. Values are represented as mean ± SE (n = 3). Different letters on columns indicate significant difference at $P < 0.05$ according to the Duncan's multiple range test.

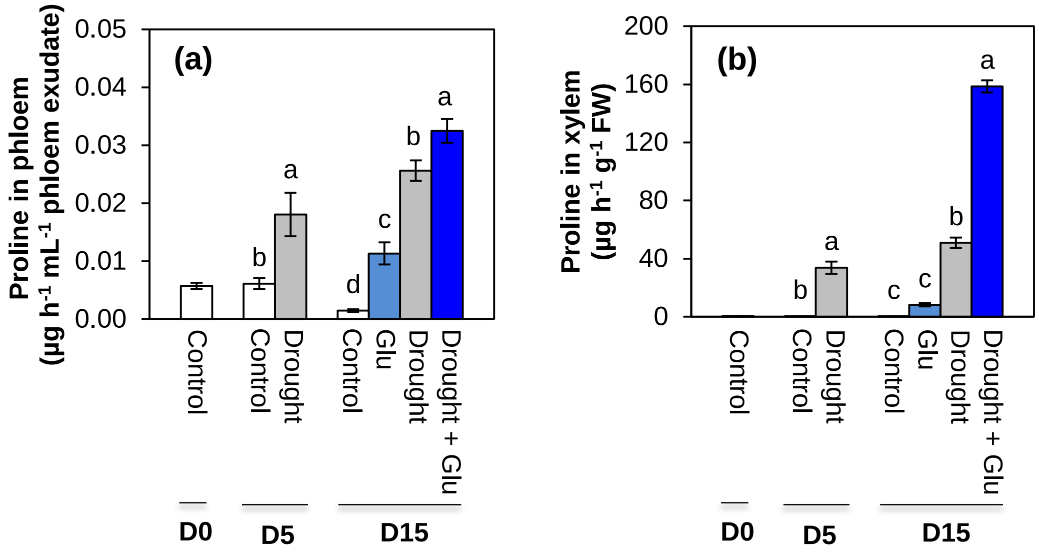

Figure S2. Effect of glutamate (Glu) application on the proline content in phloem and xylem in *Brassica napus* under well-watered or drought-stressed condition. Proline content in (a) phloem exudates and (b) xylem sap. Values are represented as mean ± SE (n = 3). Different letters on columns indicate significant difference at $P < 0.05$ according to the Duncan's multiple range test.
