# Supplementary material for: Characterization of Glutamate-Mediated Hormonal Regulatory Pathway of the Drought Responses in Relation to Proline Metabolism in Brassica napus L
Source: Plants (Basel). 2020 Apr 16;9(4):512. doi: 10.3390/plants9040512 (PMC7237994; doi:10.3390/plants9040512)
Supplement: Supplementary file 1 [file plants-09-00512-s001.zip › Tables. Supplementary.docx]

**Table S1.** Oligonucleotide primer sequences used for quantitative real-time PCR

| **No.** | **Name** | **Accession No.** | **Forward sequence of primers (5′-3′)** | **Reverse sequence of primers (5′-3′)** | **Tm (°C)** |
| --- | --- | --- | --- | --- | --- |
| 1 | Actin | AF111812.1 | F: 5′-GATTCCGTTGCCCTGAAGTA-3**′** | R: 5**′**-GCGACCACCTTGATCTTCAT-3**′** | 56 |
| 2 | BnICS1 | XM_013845172.1 | F: 5**′**-TCAATCCCAGAACGAGATCC-3**′** | R: 5**′**-GACAGAAACCTTCGGATGGA-3**′** | 56 |
| 3 | BnNPR1 | EF613226 | F: 5**′**-TGAGAACATTGCCAAGCAAG-3**′** | R: 5**′**-CAACAGCAAAATGGAGAGCA-3**′** | 56 |
| 4 | BnPR1 | XM_013826324 | F: 5**′**-GAGTAGCGCCGACTTTTCTG-3**′** | R: 5**′**-TTTGCCACATCCAATTCTCA-3**′** | 56 |
| 5 | BnMYB2.1 | JN379095 | F: 5**′**-GGAATCGATCCAACCAATCA-3**′** | R: 5**′**-AGACCAATGTTGAGGTCAGG-3**′** | 56 |
| 6 | BnNAC55 | NM_001315826.1 | F: 5**′**-CGGGTTTAACCGAACAGAAA-3**′** | R: 5**′**-TGTTGCTGCGTCTTATCGTC-3**′** | 56 |
| 7 | BnNADPH oxidase | XM_013847449.1 | F: 5**′**-CACCTCTCCCTCTTTCTGT-3**′** | R: 5**′**-CGTTGGGGTTTTGTCGCTAT-3**′** | 58 |
| 8 | BnCPK5 | JX122911.1 | F: 5**′**-TGGAAGCGTGTCATTCTCTG-3**′** | R: 5**′**-TATAACACCAGCGGTCCACA-3**′** | 57 |
| 9 | BnWRKY28 | NM_001315647.1 | F: 5**′**-GCCAGAGGAAACGAGAGTTG-3**′** | R: 5**′**-TATTTTTGAAGGCCTTTTGG-3**′** | 54 |
| 10 | BnCAT | JN163870 | F: 5**′**-GATCCTGCGGATGAGGATAA-3**′** | R: 5**′**-AAGCAGCTTGTCATCCGAGT-3**′** | 56 |
| 11 | BnTRXh5 | XM013811982 | F: 5**′**-GAAGTCTGGAGCGAGAAGAT-3**′** | R: 5**′**-CGATCTTGAAGAAGACAACG-3**′** | 54 |
| 12 | BnGRXC9 | XM013875950 | F: 5**′**-GTAACCCCAGCGGTTCTTGA-3**′** | R: 5**′**-ACCACAAAGCTCCAACATCC-3**′** | 57 |
| 13 | BnTGA2 | XM_013887736.2 | F: 5**′**-CGTGCTTTACTTGCGATTCA-3**′** | R: 5**′**-TTTCCTGGTTTGTGTGTCCA-3**′** | 56 |
| 14 | BnP5CS1 | AF314811 | F: 5**′**-CGATTTGGACTTGGTGCTGA-3**′** | R: 5**′**-GCCCATCCTCTCCTAGTC-3**′** | 56 |
| 15 | BnP5CS2 | AF314812 | F: 5**′**-CCATTATCTTCCTCCTCTCAC-3**′** | R: 5**′**-AACAACTGCTGTCCCAACC-3**′** | 56 |
| 16 | BnP5CR | XM013812259 | F: 5**′**-TTCAGTAATGAGCCTTGGAA-3**′** | R: 5**′**-TCTGTGAAGCTAAACCCAAA-3**′** | 54 |
| 17 | BnPDH | EU375567 | F: 5**′**-CGATTTGGACTTGGTGCTGA-3**′** | R: 5**′**-GCCCATCCTCTCCTAGTC-3**′** | 56 |
| 18 | BnP5CDH | XM013787440 | F: 5**′**-CTACGAACTCGTCAC CAAAG-3**′** | R: 5**′**-AGGTCCAAACCAATGATTCT-3**′** | 55 |
| 19 | BnGLR1.3 | XR_002655614.1 | F: 5**′**-TGCTGGTGATAACCGTCAAG-3**′** | R: 5**′**-GAACCCACTCCACTGCTGTT-3**′** | 56 |
